# Heterogeneously integrated light emitting diodes and photodetectors in the metal-insulator-metal waveguide platform

**DOI:** 10.1515/nanoph-2022-0784

**Published:** 2023-05-03

**Authors:** Kyungmok Kwon, Junghoon Park, Jong-Bum You, Kyoungsik Yu

**Affiliations:** School of Electrical Engineering, Korea Advanced Institute of Science and Technology (KAIST), 291 Daehak-ro, Yuseong-gu, Daejeon 34141, Republic of Korea; Department of Electrical Engineering and Computer Sciences, University of California, Berkeley, CA 94720, USA; Department of Nanodevice Technology, National Nanofab Center (NNFC), 291 Daehak-ro, Yuseong-gu, Daejeon 34141, Republic of Korea

**Keywords:** compound semiconductor, light emitting diodes, metal-insulator-metal waveguides, photodetectors, spontaneous emission

## Abstract

We demonstrate heterogeneous integration of active semiconductor materials into the conventional passive metal-insulator-metal (MIM) waveguides to provide compact on-chip light generation and detection capabilities for chip-scale active nanophotonic platforms. Depending on its bias conditions, a metal-semiconductor-metal section can function as either a light emitting diode or a photodetector directly connected to the MIM waveguides. We experimentally verify the independent and combined operations of electrically-driven on-chip light sources and photodetectors.

## 1 Introduction

A simple one-dimensional stack of metal-insulator-metal (MIM) layers can strongly confine the electromagnetic fields between two metal cladding layers, and therefore allows the realization of various photonic devices and systems at the wavelength- and subwavelength-scale physical dimensions [1–7]. Due to their performances and simplicity, the MIM waveguide geometries have been very successful nanophotonic platforms for a number of applications, such as nanoscale light focusing [1], interferometry [2, 3], resonators [3, 4], and photonic circuits [5–7]. However, in most cases, the light excitation and detection for such miniaturized MIM platforms have relied upon non-nanophotonic, external devices and systems, such as near-field scanning optical microscopes (NSOMs) [8] as well as external light sources and photodetectors. For example, external laser illumination is often coupled to surface plasmon polaritons via a grating coupler to overcome the momentum mismatch [9], and the focused and guided electromagnetic field distribution can be observed by an NSOM [8, 9]. Electrically-driven on-chip light sources (on-chip electrical-to-optical conversion) and integrated photodetectors (on-chip optical-to-electrical conversion) may significantly alleviate the necessity of such external devices for optical excitation and detection, and can play critical roles for a number of practical nanophotonic applications, such as chip-scale optical interconnects, bio/chemical detection, and data storage [10–19].

**Figure 1:**
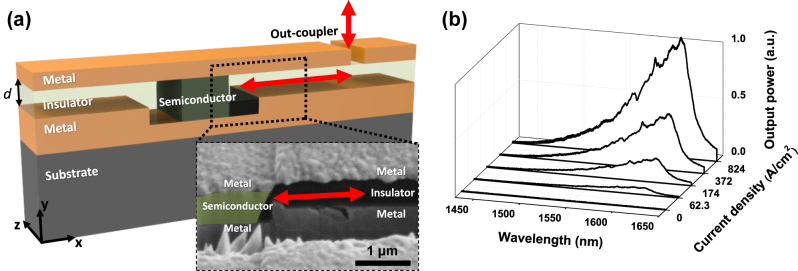
Schematic diagram and electroluminescence of an MSM section embedded in the MIM waveguide. (a) Schematic showing an integrated, electrically-driven active nanophotonics platform composed of a metal-semiconductor-metal active section and metal-insulator-metal waveguide. The inset indicates a cross-sectional scanning electron micrograph (SEM) of a fabricated device, corresponding to the dashed box area in the schematic. The BCB thickness, *d*, was designated close to ∼500 nm. The scale bar in the SEM represents 1 μm. (b) The electroluminescence spectra are shown with respect to various current densities under the positive applied bias.

Regarding the electrically-driven on-chip light sources, there are many potential mechanisms to realize light emission. Inelastic tunneling, for example, involves the transfer of energy from an injected electron to a localized plasmon mode, which then decays radiatively to produce light [16, 17]. Luttinger liquid plasmons are collective excitations of the electron liquid in one-dimensional systems and can also be used as a light source with strong confinement in nanometer-scale structures. While these mechanisms have the potential to offer unique advantages in certain applications such as spectroscopy and sensing, they may require high electric fields, deep subwavelength-scale low-dimensional structures, and sometimes low temperatures, which can limit their practical use in on-chip light sources. In our work, we chose the semiconductor-based bandgap emission mechanism because of its high conversion efficiency, and compatibility with the heterogeneous integration of the MIM section. This mechanism involves the recombination of electron-hole pairs in a semiconductor material with a specific bandgap energy. Specifically, we utilized a p-i-n InAlGaAs/InGaAs/InAlGaAs heterostructure with highly doped cladding layers to improve carrier confinement and radiative recombination rate. One important advantage of using a direct-bandgap semiconductor heterostructure is that its function can be easily switched between electrical-to-optical and optical-to-electrical conversion simply by changing the bias polarity. While the concept of on-chip light generation and detection has been explored in the past, these have mostly been investigated separately. There are only a few examples of on-chip light emitters and photodetectors implemented together [17–19]. Despite the necessity of on-chip photonic systems, the difficulty of heterogeneous material integration has limited the intimate integration of various optical components, including light sources, detectors, and waveguides [20, 21]. The metal-semiconductor-metal (MSM) multilayer stack is a promising platform for intimate chip-scale integration of both active and passive photonic/optoelectronic blocks because the metal cladding layers can not only confine the optical/plasmonic modes similarly with the conventional MIM waveguide geometries but also function as electrodes to the active semiconductor devices. In the MSM/MIM platform, semiconductor materials are intimately embedded in the MIM waveguides, and therefore the electrically-driven semiconductor-based light sources and photodetectors are directly embedded into the MIM waveguides as schematically described in Figure 1(a). In this work, we investigate heterogeneous integration of MSM active devices, such as light emitting diodes (LEDs) and photodetectors (PDs), within the conventional MIM waveguide geometries, enabling compact and efficient on-chip light generation and detection for chip-scale active nanophotonic platforms.

## 2 Results & discussion

### 2.1 Integration of light emitting diodes within the MIM waveguide

For heterogeneous integration of active semiconductor-based optoelectronic devices in a conventional vertically-stacked metal-optic platform, a p-i-n semiconductor heterostructure with direct top and bottom metal contact electrodes is placed into the MIM waveguide as shown in Figure 1(a). The MIM waveguide section consists of the vertical stack of Ag (200 nm)/BCB (benzocyclobutene, *d* = 500 nm)/Ag (>>200 nm) layers, and the MSM region contains Ag (200 nm)/a middle InGaAs active layer with InAlGaAs cladding layers (540 nm)/Ag (>>200 nm). Since the subwavelength-scale MSM/MIM multilayer thin film stack is bonded on the silicon substrate using thermocompression metallic bonding, the thickness of the bottom metal layer is much thicker than 200 nm. Thin titanium layers were used as an adhesion layer for the Ag metal thin films. The detailed fabrication process can be found in our previous works [22, 23] and Supplementary Figure S1.

In order to improve the radiative recombination rate as well as carrier confinement, the p-i-n InAlGaAs/InGaAs/InAlGaAs heterostructure was utilized [24] with the upper and lower InAlGaAs cladding layers highly doped at the volume concentrations of 2 × 10^19^ cm^−3^ and 5 × 10^19^ cm^−3^ for the p- and n-type doping, respectively. Table S1 describes the information of p-i-n InAlGaAs/InGaAs/InAlGaAs epitaxial layers. The inset of Figure 1(a) shows the cross-section of a fabricated sample at the interface between the active MSM region and the MIM waveguide on the silicon substrate. Red arrows in Figure 1(a) schematically represent the light propagation directions. To observe the guided light emission from the semiconductor’s active region through the MIM waveguide, rectangular light emission slits were milled by a focused ion beam (FIB) at the 200 nm-thick top metal layer. A single out-coupling slit is schematically described in Figure 1(a). The same FIB milling process can be used to define the electrical isolation between the top electrodes when multiple active MSM devices are integrated on the common bottom metal layer.

The electrical characteristics of the fabricated MSM structures were consistent with the typical behavior of pn junction diodes as shown in Supplementary Figure S2. Under the forward bias conditions, the current flow exponentially increases with the applied bias voltage, whereas the limited reverse current flow is observed due to the rectifying characteristics of typical pn junctions. As shown in Figure 1(b), we observe electroluminescence spectra under the forward bias conditions, and the light output power grows in proportional to the input current density as in typical LEDs. In order to measure the emission spectra, we used a microscope-based electroluminescence setup. While the current–voltage characteristics were recorded using a source measure unit (Keithley 236), vertically scattered light emission was collected through a microscope objective with a numerical aperture of 0.5. The output light spectra were analyzed by an infrared spectrometer with a linear photodetector array (iDus InGaAs 1.7, Andor).

**Figure 2:**
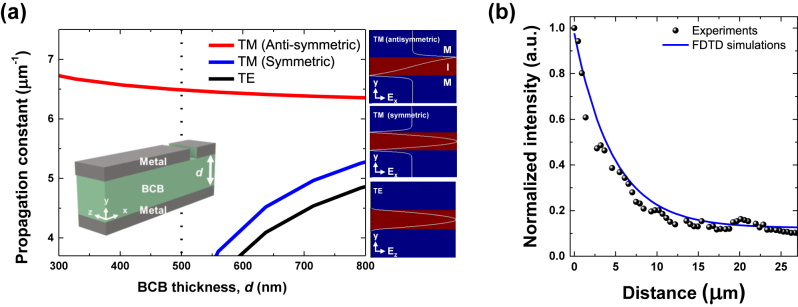
MIM waveguide’s propagation constant and loss. (a) Propagation constant as a function of the BCB thickness, *d,* at a free-space wavelength of 1550 nm. Mode profiles of each mode supported in the MIM waveguide: Antisymmetric TM, symmetric TM, and TE mode. When *d* < 550 nm, the antisymmetric TM mode is guided while symmetric TM mode and TE mode are neatly cut-off. (b) Intensity of scattered light at out-coupling slit as a function of distance from the active MSM region. The measured exponential decaying behavior well corresponds to the two-dimensional FDTD simulation results.

To investigate the propagation modes for the one-dimensional vertical MIM stack, we used a two-dimensional eigenmode expansion solver (MODE, Lumerical) with an assumption that the metal cladding layers are sufficiently thick. For the material parameters in the calculations, we used the data from Palik [25]. More specifically, the refractive indices of BCB and Ag are 1.5 and 0.469 + 9.32i at 1550 nm, respectively. Without loss of generality, the wave propagation occurs along the horizontal axis (*x*-axis). In Figure 2(a), we show the propagation constants of several low order modes as a function of the insulator or dielectric layer (BCB material in our implementation) thickness, *d*, at a representative wavelength of 1550 nm for this planar MIM waveguide geometry. The propagation mode with the largest propagation constant is the antisymmetric transverse magnetic (TM) mode with an antisymmetric *E*_*x*_ field profile across the vertical *y*-axis as shown in the upper right panel of Figure 2(a). The electric field profiles for other modes (symmetric TM mode and the transverse electric (TE) mode) are also shown in Figure 2(a). When the thickness of the dielectric BCB layer is below 550 nm (*d* < 550 nm), the TE and symmetric TM modes are nearly cut off, but the antisymmetric TM mode is maintained. This means that the output light from the MSM region can be coupled to only the antisymmetric TM mode within the emission bandwidth of InGaAs LEDs near 1550 nm when *d* = 500 nm.

In order to investigate the light propagation distance and attenuation along the fabricated MIM waveguide (*d* = 500 nm), out-coupling slits were patterned at various distances from the MSM active region, and the light output intensities were quantitatively measured by a two-dimensional infrared photodetector array through a microscope. An example of an out-coupling slit with a thin rectangular aperture perpendicular to the light propagation direction is schematically described in Figure 1(a). Owing to the ohmic losses in the metal cladding layers of the MIM waveguide, the output intensities decay exponentially with respect to the propagation distance as observed in Figure 2(b). A good agreement between the observed and calculated decay lengths was obtained when the effects of the titanium adhesion layer was considered. The simulations were performed using two-dimensional FDTD methods. More simulation results on the propagation losses can be found in Figure S4. Since the propagation constant of the antisymmetric TM mode for the free-space wavelength of 1550 nm is approximately 6.5 μm^−1^, its effective wavelength along the MIM waveguide is less than 1 μm. The two-dimensional simulation results are valid in the regime where the beam width (along the *z* axis) is sufficiently larger than the effective wavelength. For the experiments, slit lengths of at least 10 μm were used.

Although the propagation distance of the MIM waveguide is shorter than that of typical dielectric waveguides, it can still be useful in on-chip optical transmission applications where compactness is a priority and there is sufficient power budget. The propagation distance of approximately 15 μm for the MIM waveguide shown in Figure 2(b) is specific to the implementation used in this study and may vary depending on the specific MIM waveguide geometry and materials used. The propagation distance can be improved by employing metal layers with lower optical losses [26]. For example, instead of the titanium adhesion layer, a germanium adhesion layer can be employed for reduced optical losses. We also observe that the output light from the out-coupling slit is strongly polarized perpendicular to the slit’s long axis (or parallel to the light propagation direction through the MIM waveguide (*x* axis)) as expected from the TM mode profile with non-zero *E*_*x*_ field components shown in Figure 2(a). This also confirms that the light output from the active MSM region is not coupled to the TE mode with negligible electric field components along the light propagation direction.

### 2.2 Integrated photodetectors

**Figure 3:**
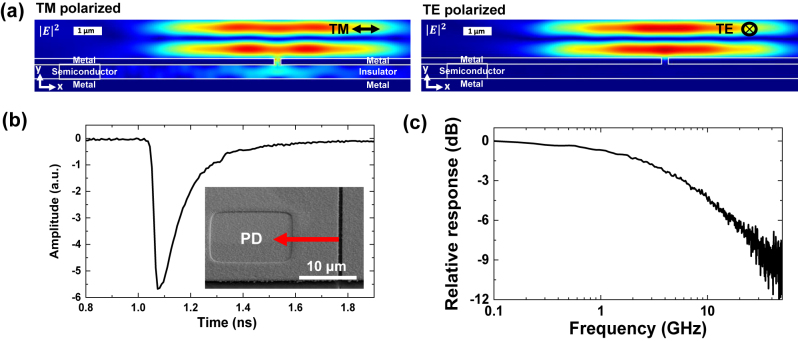
Simulation and measurement results for integrated PDs. (a) The calculated electric field intensities for a MIM-MSM device are shown in both TE-polarized and TM-polarized input cases. The normally incident light efficiently couples to the MIM waveguide for the TM polarization case only. The input Gaussian beam size is 10 μm, and the BCB thickness is *d* = 500 nm. (b) and (c) High-speed integrated photodetector. (b) An example impulse response is shown for a PD at the bias of −0.3 V. The inset shows the SEM of the PD device used in the experiments. The scale bar represents 10 μm. (c) The frequency response obtained from the Fourier transform of the impulse response in (b) indicates the 3 dB bandwidth of ∼6 GHz.

Under the reverse bias conditions, the MSM semiconductor-based light source shown in Figure 1(a) functions as a PD directly integrated with the MIM waveguide. Two-dimensional FDTD simulations and experimental results support the polarization-dependent operation of the integrated PD as described in Figure 3. For simulations, a finite Gaussian beam at a free-space wavelength of 1550 nm with a TM (electric field parallel to the *x*-axis) or TE (electric field parallel to the *z*-axis) polarization state was illuminated from the surface normal direction (propagating along the negative *y*-axis) toward the out-coupling slit whose long axis is along the *z*-axis, and the normalized electric field intensity profile was depicted. As expected, the incident TE-polarized wave is not coupled to the MIM waveguide. In case of the TM polarization, however, the illuminated wave first excites a gap plasmonic mode at the rectangular aperture, and then gets coupled to the propagating antisymmetric TM mode of the MIM waveguide. The coupled and propagated wave through the MIM waveguide is finally absorbed in the semiconductor block of the MSM section because its high refractive index leads to strong modal confinement and absorption between two metal planes. For experimental verification, linearly polarized laser light with a wavelength of 1520 nm was illuminated, and the polarization-dependent photocurrent generation was observed.

The operation bandwidth of the integrated PD was investigated from its impulse response. A picosecond mode-locked laser with a pulse width of ∼0.5 ps (PriTel, Inc.) and a wavelength of ∼1550 nm was used as an input light source. The polarized pulsed light input was converted to the photocurrent through the embedded PD in the MSM section as described above, and the output signal was observed on an oscilloscope. Figure 3(b) presents an example of representative impulse responses measured at the reverse bias of −0.3 V for an MSM active region area of 15 μm × 15 μm. The rising and falling time are observed as 21 ps and 220 ps, respectively. Although the calculated 3 dB bandwidth from the Fourier transform of this impulse response was ∼6 GHz as shown in Figure 3(c), it could have been as large as 17.5 GHz when we consider the rising time only (∼0.35/*τ*_*r*_ for a rising time-limited bandwidth).

The overall bandwidth of typical p-i-n PDs is limited by both the carrier transit time and the RC time constant. When considering an MSM active area of 15 μm × 15 μm, the PD bandwidth from the RC delay time is estimated to be >100 GHz and therefore it is not a dominant factor that limits the overall PD bandwidth. For experimental verifications, various square-shaped MSM structures with side dimensions of 20 µm, 6 µm and 2 µm were also fabricated and compared. Dimension-independent PD bandwidths according to the normalized impulse responses in Figure S3 confirms that the PD bandwidth is indeed not constrained by the RC time constant but by the carrier transit time, especially because of the carrier diffusion tail and trapping observed in the slow falling edge of the impulse response in Figure 3(b). Due to the antisymmetric TM mode profiles, strong light intensities near the highly-doped semiconductor cladding layers result in non-negligible light absorption in the p-i-n PD’s diffusion region, which causes the long minority carrier diffusion tail [27]. We believe that the overall PD bandwidth can be further improved by proper epitaxial layer designs.

### 2.3 Chip-scale integration of light sources and photodetectors

**Figure 4:**
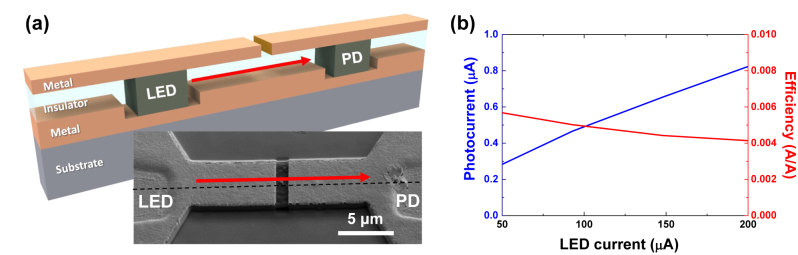
Electrically-driven light source and photodetector integrated in the MIM waveguide platform. (a) Simultaneous operation of multiple MSM sections connected with a one-dimensional MIM waveguide. The demonstrated electrically-driven optical link consists of an LED, MIM waveguide, and a PD. An SEM of the demonstrated optical link is shown in the inset. A slot in the middle of the link was ion-milled by FIB for electrical isolation. The scale bar represents 5 μm. (b) Photocurrent and system efficiency as a function of LED drive current. The gradual decline of overall link efficiency attributes to the reduced quantum efficiency of the LED section.

To experimentally demonstrate intimate chip-scale integration of both light sources and photodetectors based on the heterogeneously integrated MSM stacks in the common MIM geometries, we fabricated a simple, short one-dimensional back-to-back link between an LED and a PD with the passive MIM waveguide as shown in Figure 4(a). The distance between the LED and the PD was ∼20 μm. For simultaneous operation of both devices, top electrodes of two MSM regions were oppositely biased, while the common bottom metal layer functions as the electrical ground. As described in the previous sub-sections, the MSM region for the LED is forward-biased for light emission, and the other MSM section is reverse-biased for photodetection operation. The spontaneously emitted light from the forward-biased MSM section is directly coupled to the one-dimensional MIM waveguide, and the propagated light is converted to the photocurrent at the other MSM section. Although vertical out-coupling is not necessary to establish this on-chip photonic link, the top metal cladding layer must have at least one discontinuity due to the electrical isolation between the oppositely biased MSM sections. Although some lights are scattered at this electrical isolation slit in the middle of the link, most of the propagated wave through the MIM waveguide is transmitted toward the PD (Figure S5).

Due to the on-chip electrical-to-optical and optical-to-electrical conversion processes, the input and output signals for the on-chip optical link are the drive current to the LED (forward-biased MSM section) and the photocurrent from the PD (reverse-biased MSM section), respectively. Figure 4(b) shows the output photocurrent with respect to the LED input current as well as their ratio. Although the PD photocurrent monotonically increases with the LED drive current, the overall conversion efficiency decreases with the LED current. Although the exact reason is not experimentally verified, the gradual reduction in the link efficiency at high LED currents may be due to the LED’s reduced quantum efficiency, possibly caused by Auger recombination and thermal stress [28–30]. At a current of 200 μA, the corresponding current density reaches ∼800 A/cm^2^ for the MSM area of 5 μm × 5 μm. At such high current density levels, nonradiative Auger recombination, which increases with the cube of the carrier density, can become more dominant and reduces the LED’s overall quantum efficiency. Furthermore, high current densities increase the LED temperature and cause thermal stress in the device, which can further reduce its performance.

The overall link efficiency is ∼0.005 A/A. One reason for the relatively low link efficiency is the low coupling efficiency between the MSM section and the MIM waveguide. The coupling efficiency can be affected by many factors, such as the thickness, position, and refractive indices of the semiconductor light emission layer within the MIM waveguide, the wavelength and polarization of light. The MSM LED emits light to multiple modes, while the MIM waveguide only supports several modes. As shown in the subpanel of Figure 2(a), the mode profile for the antisymmetric TM mode, which has the lowest propagation loss, has higher intensity distribution near the semiconductor(insulator)-metal interfaces. However, the active semiconductor region is located in the middle of the waveguide to provide the Ohmic contacts through the metal cladding layers, resulting in low spatial overlap with the antisymmetric TM mode. As a result, only a small fraction of the light emitted from the LED will couple to the waveguide propagation mode. This inefficiency in coupling also applies to the photodetector parts. The link efficiency can be further improved by using more advanced designs for mode coupling structures between the MSM sections and the MIM waveguides. The epitaxial layers can also be modified to better match the propagation mode, and enhance the Purcell factor. Although the Purcell enhancement has not been experimentally proven in our proof-of-concept MIM/MSM integration scheme, we believe that an improved adhesion layer and epitaxial layer design providing optical gains close to the metal-semiconductor interface may significantly improve the overall efficiency of light generation and detection in the MSM section.

## 3 Conclusions

In summary, we have successfully demonstrated heterogeneous integration of multiple MSM sections with compound semiconductor materials embedded within the conventional MIM waveguide geometries, and verified the independent and combined operation of integrated on-chip light sources and detectors. The multiple MIM/MSM sections and their circuits can be batch-fabricated from the epitaxially-grown compound semiconductor substrate, and then transferred to another substrate, such as the silicon substrate as demonstrated in this work, through the thermocompression bonding processes. The proposed MSM/MIM platform allows intimate integration of passive MIM nanophotonic components and semiconductor-based active optoelectronic devices, such as LEDs and PDs, and thereby enables compact realization of interesting photonic devices and systems. For example, an MIM metasurface with fishbone patterns on the top metal layer can be directly integrated with an underlying semiconductor PD to realize a compact, integrated polarization-state analyzer [31].

Electrically-pumped on-chip amplifiers and lasers at room temperature are desirable because they can be intimately integrated in photonic integrated circuits for a number of applications [15]. However, there are several challenges to overcome, such as high ohmic losses in metals, the trade-off between confinement and gain, and the fabrication of nanoscale structures. Recent advances in materials engineering, nanofabrication techniques, and theoretical modeling have shown some possible solutions to these problems, such as using hybrid metal-semiconductor structures, optimizing the geometry and dimensions of the plasmonic cavities, and exploiting Purcell enhancement and tunneling injection. With improved epitaxial semiconductor layer designs that can improve the spatial modal overlap and provide more optical gains at reduced carrier densities, it might also be possible to provide on-chip optical gains to the propagating MIM modes, enabling even more functionalities to the proposed MIM/MSM active nanophotonics platform for a number of emerging applications [32–34].

## Supplementary Material

Supplementary Material Details
