# Supplementary material for: Heterogeneously integrated light emitting diodes and photodetectors in the metal-insulator-metal waveguide platform
Source: Nanophotonics. 2023 May 3;12(13):2603–10. doi: 10.1515/nanoph-2022-0784 (PMC11501676; doi:10.1515/nanoph-2022-0784)
Supplement: Supplementary file 1 — Supplementary Material Details [file j_nanoph-2022-0784_suppl_001.pdf]

Heterogeneously integrated light emitting diodes and photodetectors in the metal-insulator-metal waveguide platform

Kyungmok Kwon,[1,2,#] Junghoon Park,[1,#] Jong-Bum You,[3] and Kyoungsik Yu[1]*

[1] School of Electrical Engineering, Korea Advanced Institute of Science and Technology (KAIST), 291 Daehak-ro, Yuseong-gu, Daejeon 34141, Republic of Korea
[2] Department of Electrical Engineering and Computer Sciences, University of California, Berkeley, CA 94720, USA
[3] Department of Nanodevice Technology, National Nanofab Center (NNFC), 291 Daehak-ro, Yuseong-gu, Daejeon 34141, Republic of Korea

* Corresponding author e-mail: ksyu@kaist.edu
# These authors equally contributed to this work.

1. Fabrication process

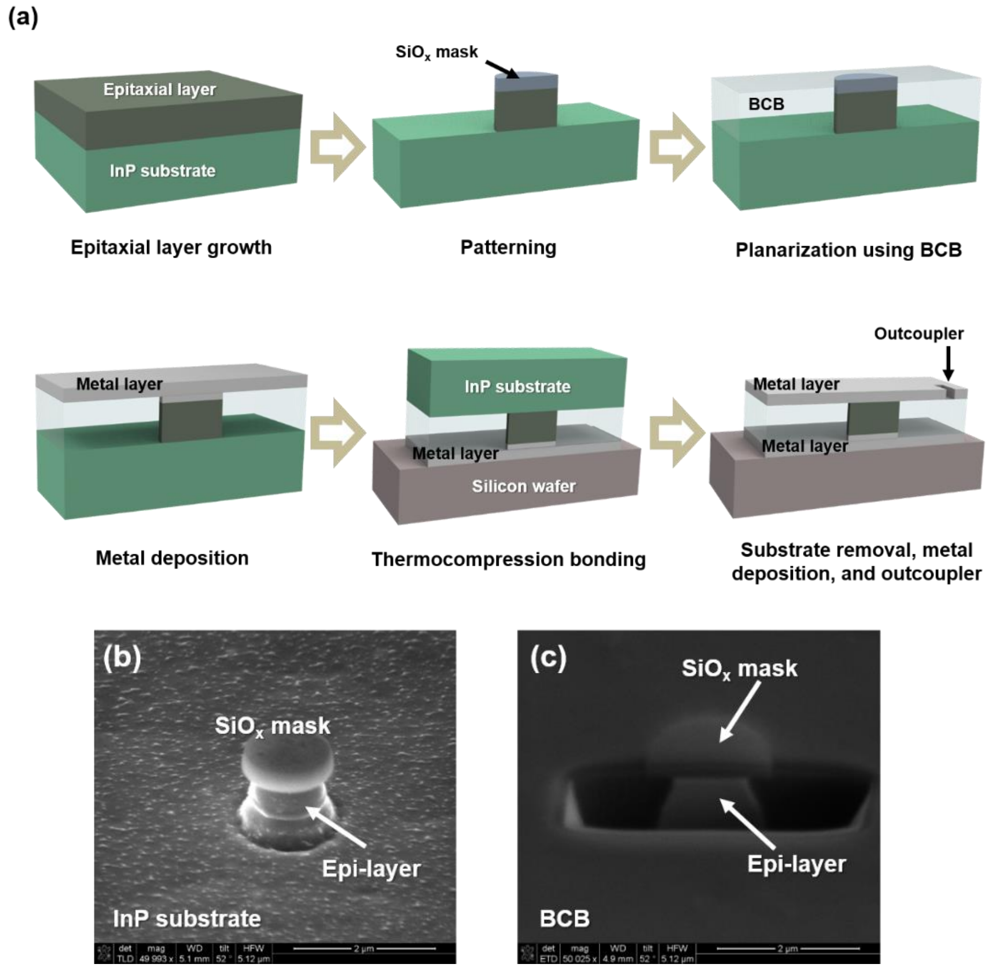

Figure S1. Sample fabrication process. (a) Detailed fabrication process steps. (b) A scanning electron micrograph (SEM) of an etched epitaxially-grown semiconductor structure after RIE and HBr:H_3PO_4:(0.5M)$K_2Cr_2O_7$ wet etching. The SiO_x hard mask is used for RIE. (c) An SEM of an etched structure after the planarization process using the BCB layer. The SiO_x hard mask is not yet removed, and can still be observed.

Figure S1(a) depicts the overall fabrication process. The p-i-n heterostructure was first grown by molecular beam epitaxy on the InP substrate. Table S1 describes the information of epitaxial layers in detail. A 300-nm-thick SiO_x hard mask layer was deposited on top of the epitaxial layer, and the semiconductor pattern was defined by electron beam lithography with a negative electron beam resist material, and then subsequently transferred to the SiO_x layer by dry etching. The epitaxial layer was then etched by reactive-ion etch (RIE). We used HBr-based RIE (instead of CH_4-based RIE) to prevent unwanted polymer formation on the sidewall and

etch mask. After RIE patterning, the sample surface was further slightly etched with a 1:1:1 HBr:H_3PO_4:(0.5M)$K_2Cr_2O_7$ isotropic etchant to remove the damaged semiconductor layer. Figure S1(b) shows an example of an etched structure with a diameter of <1 μm after RIE and HBr:H_3PO_4:(0.5M)$K_2Cr_2O_7$ wet etching. The semiconductor structures used in this work were larger than this structure. BCB was spin-coated on the sample, and the coated BCB layer was etched back to expose the SiO_x hard mask as shown in the planarization step of Figure S1(a). Figure S1(c) shows an example of a planarized etched structure at this step.

After planarization, the oxide hard mask was removed by chemical etching, and subsequently multiple metal layers (Pt/Ti/Ag/Pt/Ag) were evaporated by an electron beam evaporator. Each layer was chosen for the p-contact, adhesion layer, low optical loss, diffusion barrier and metallic bonding layer, respectively. Once the metal layers were evaporated, the sample was flipped and bonded to another silicon handle wafer via thermocompression metallic bonding cycle with BiSnAg solder foil, and then the InP substrate side was mechanically ground by a lapping machine to reduce the InP substrate thickness. The sample was then placed into a HCl:H_3PO_4 acid solution to completely remove the remaining InP substrate. Finally, the top metal electrode patterns were defined by a simple lift-off process with Ti/Ag n-contact metal layers . Fine light out-coupling slit patterns (rectangular apertures) were finally formed by a focused ion beam milling.

Table S1. Epitaxial layer information. (The substrate is at the bottom of the layer 1.)

	**Layer**	**Material**	**Thickness (Å)**	**Doping level (cm^{-3})**	**Type**
9	Capping layer	$In_{0.53}Ga_{0.47}As$	1,000	2.0E+19	p++
8	Contact	InP	400	1E19	p++
7	Optical confinement	$In_{0.53}Al_{0.279}Ga_{0.721}As$	400	2.0E+18	p
6	Optical confinement	Graded InAlGaAs	400	Undoped	
5	Active layer	$In_{0.53}Ga_{0.47}As$	2,000	Undoped	
4	Optical confinement	Graded InAlGaAs	400	Undoped	
3	Optical confinement	$In_{0.53}Al_{0.279}Ga_{0.721}As$	200	2.0E+18	n
2	Optical confinement	$In_{0.53}Al_{0.279}Ga_{0.721}As$	200	2.0E+19	n++
1	Contact	$In_{0.53}Ga_{0.47}As$	400	5.0E+19	n++

2. Electrical characteristics of a p-i-n diode

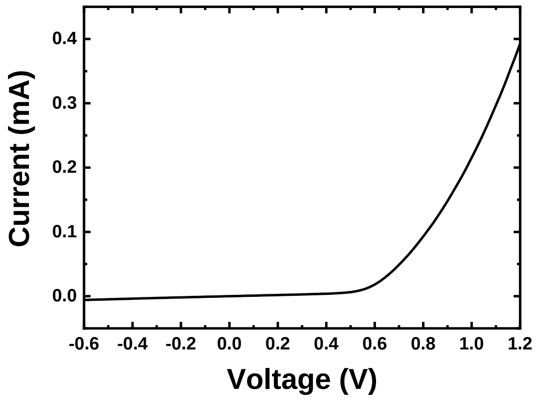

Figure S2: Electrical characteristics of a representative p-i-n diode in the MSM region. The *I-V* characteristics of a typical p-i-n device show the rectifying behavior at negative bias voltages. Under forward bias conditions, electroluminescence was observed as shown in Figure 1(b) in the main manuscript.

3. Impulse responses of various PDs with different dimensions

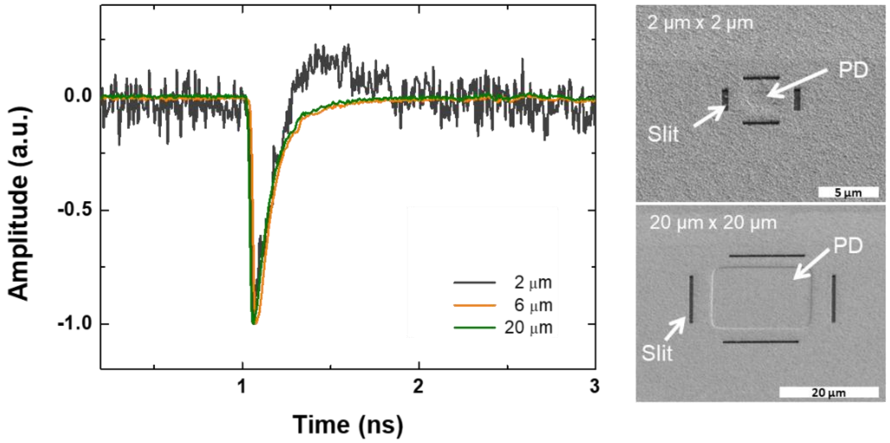

Figure S3: Impulse response of various integrated PDs with different dimensions and SEMs. The impulse responses of three square-shaped PDs (20 μm × 20 μm, 6 μm × 6 μm, and 2 μm × 2 μm) were measured, and the normalized impulse responses are plotted together. The measured rising and falling time are ~20 ps and ~200 ps, respectively, regardless of the PD dimensions. Relatively slow falling time is caused by light absorption in the thick doped layers.

4. Propagation loss of MIM waveguides

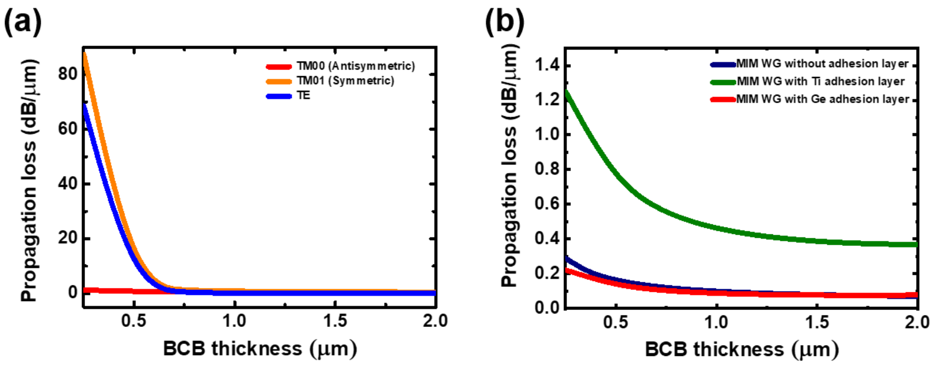

Figure S4: Propagation loss as a function of the BCB thickness, *d*. (a) Propagation loss of each mode supported in the MIM waveguide: Antisymmetric TM, symmetric TM, and TE mode. When $d < 550$ nm, the antisymmetric TM mode can still be guided with moderate propagation losses, while the propagation losses of symmetric TM and TE modes increase quickly due to cut-off. The calculation includes the effect of the titanium adhesion layer. (b) Propagation loss of the antisymmetric TM mode with and without the adhesion layer. The propagation loss significantly increases when titanium is introduced as an adhesion layer. When the Ti layer is replaced with a 10-nm-thick Ge layer, as suggested in the main manuscript, the propagation loss is significantly reduced and becomes similar to the ideal case without the adhesion layer.

5. Optical loss from electrical isolation

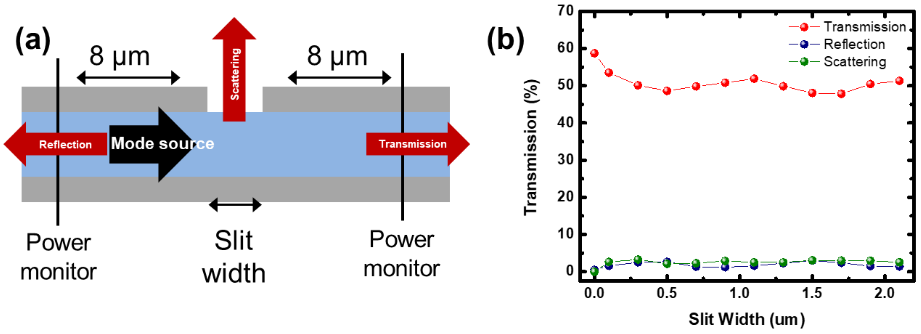

Figure S5: Transmission through the electrical isolation slit. (a) Schematic of the FDTD simulation geometry. (b) Calculated transmission, reflection, and scattering.

We calculated the transmission of the antisymmetric TM mode through the electrical isolation slit (FDTD Solutions, Ansys Lumerical). The antisymmetric TM mode was excited from the mode source, and the fraction of transmitted and reflected power was measured using a

frequency domain power monitor. To analyze the effects of the electrical isolation trench, transmission power was calculated as a function of the slit width, as shown in Figure S5 (b). The MIM propagation mode (antisymmetric TM mode) was converted to the IM mode upon its incident at the slit, and then it was coupled back to the MIM mode [S1]. Our electrical isolation slit was approximately 1 μm, and its additional loss was estimated to be approximately 3 dB (~50% transmission) due to reflection, scattering, and propagation losses of the IM mode.

Supplementary Material References

S1. Walters, R.J., et al., *A silicon-based electrical source of surface plasmon polaritons.* Nature materials, 2010. **9**(1): p. 21-25.
